# Visceral Adiposity and Markers of Relative Sarcopenia in Young Adults with Normal Weight Obesity: Gender Differences

**DOI:** 10.3390/healthcare14091243

**Published:** 2026-05-05

**Authors:** Rodrigo Yáñez-Sepúlveda, Boryi A. Becerra-Patiño, Armando Monterrosa-Quintero, Carlos Abraham Herrera-Amante, César Octavio Ramos-García, Guillermo Cortés-Roco, Exal Garcia-Carrillo, Eduardo Guzmán-Muñoz, Yeny Concha-Cisternas, José Francisco López-Gil

**Affiliations:** 1 Faculty Education and Humanities, School of Sport Sciences, Universidad Andres Bello, Viña del Mar 2520000, Chile; rodrigo.yanez.s@unab.cl; 2 Department of Sport Sciences, Faculty of Sport and Health Sciences, Fit Generation Research Institute, AD500 Andorra la Vella, Andorra; 3 Faculty of Physical Education, National Pedagogical University, Bogota 111166, Colombia; babecerrap@pedagogica.edu.co; 4 Faculty of Sport Science, University of Murcia, 30100 Murcia, Spain; 5 Altius Performance Laboratory, Physical Education and Sports Program, Universidad Surcolombiana, Neiva 410001, Colombia; adomonterrosa@gmail.com; 6 Nutritional Assessment and Nutritional Care Laboratory, Division of Health Sciences, Tonalá University Center, University of Guadalajara, Tonalá 45425, Mexico; carlos.amante@academicos.udg.mx (C.A.H.-A.); cesar.ramos@cutonala.udg.mx (C.O.R.-G.); 7 Ibero-American Network of Researchers in Applied Anthropometry, 04120 Almería, Spain; 8 Facultad de Ciencias de la Vida, Universidad Viña del Mar, Viña del Mar 2520000, Chile; guillermo.cortes@uvm.cl; 9 Department of Physical Activity Sciences, Faculty of Education Sciences, Universidad Católica del Maule, Talca 3480112, Chile; exal.garcia@gmail.com; 10 School of Education, Faculty of Human Sciences, Universidad Bernardo O’Higgins, Santiago 8370993, Chile; 11 Escuela de Kinesiología, Facultad de Salud, Universidad Santo Tomás, Talca 3460000, Chile; eguzmanm@santotomas.cl (E.G.-M.); yenyconchaci@santotomas.cl (Y.C.-C.); 12 Escuela de Pedagogía en Educación Física, Facultad de Educación, Universidad Autónoma de Chile, Talca 3460000, Chile; 13 Vicerrectoría de Investigación e Innovación, Universidad Arturo Prat, Iquique 1100000, Chile; 14 School of Medicine, Universidad Espíritu Santo, Samborondón 092301, Ecuador; 15 Vicerrectoría de Investigación y Postgrado, Universidad de Los Lagos, Osorno 5290000, Chile

**Keywords:** obesity paradox, body composition, relative sarcopenia, public health

## Abstract

**Background and Objective**: Body mass index (BMI) is a worldwide screening standard but fails to distinguish between fat mass and fat-free mass. This study examines the prevalence and metabolic profile of the normal weight obesity (NWO) phenotype in a large cohort of young adults. **Methods**: A cross-sectional study of 4793 young adults (18–35 years) was conducted using bioelectrical impedance analysis (BIA). Participants were stratified into four phenotypes: underweight, healthy weight (HW), NWO, and obesity. Anthropometric indices, visceral fat area (VFA), and phase angle (PhA) were analyzed. **Results**: Within the normal BMI range (*n* = 2491), 40.6% (*n* = 1012) of patients were classified as NWO (percentage of body fat (PBF) >30% for women, >20% for men). NWO subjects showed a significantly higher VFA compared to the HW (+33.0 cm^2^ in men and +24.3 cm^2^ in women; *p* < 0.001) with a very large effect size (Cohen’s d > 2.0). Furthermore, a state of relative sarcopenia was identified, characterized by significantly lower skeletal muscle mass percentage (SMM%) and PhA (*p* < 0.001; d = −0.82), indicating compromised cellular integrity despite a normal BMI. **Conclusions**: BMI misclassifies 4 out of 10 young adults with excess adiposity. NWO is a high-risk phenotype linked to visceral adiposity and early cellular frailty. Incorporating BIA in routine screenings is essential to identify this invisible risk group.

## 1. Introduction

According to the World Health Organization (WHO), the global prevalence of obesity has reached epidemic proportions; by 2020, one in eight people worldwide was living with obesity, representing a twofold increase among adults and a fourfold increase among adolescents since 1990 [1]. In that same year, approximately 2.5 billion adults (43%) were classified as overweight, including 890 million (16%) with obesity [2]. The WHO clinically defines overweight as a body mass index (BMI) of 25.0–29.9 kg/m^2^ and obesity as a BMI of ≥30 kg/m^2^, both of which reflect health risks derived from excess adiposity [1]. This excessive fat accumulation is a major driver of global morbidity and mortality, associated with a reduction in life expectancy of 5 to 20 years depending on severity and coexisting conditions [3,4]. However, cardiometabolic risk is not confined to elevated BMI ranges. Normal-weight obesity (NWO) describes a high-risk profile characterized by abundant adiposity despite maintaining a normal body weight [5]. Alarmingly, NWO is strongly associated with severe physiological impairments, including insulin resistance, oxidative stress, chronic low-grade inflammation, and elevated cardiovascular risk [6,7].

In the last few years, the life expectancy of the global population has been increasing as compared to the last decade [8]. However, obesity is now an indicator of morbidity and mortality risk in different population groups, which is especially concerning young people and dangerously so in older adults [9]. A previous study that analyzed variables related to age and body fat distribution showed that overweight and obesity increased steadily, with a decrease after the age of 70 [10]. Meanwhile, visceral obesity has been defined as an excessive increase in adipose tissue near the intra-abdominal organs [11]. One of the difficulties associated with visceral obesity is the decrease in the regulatory processes of pro-inflammatory responses [12]. This means that excessive growth of visceral adipose tissue leads to high metabolic risk [13] and hemodynamic risk linked to coronary heart disease, kidney problems [14], and other issues associated with hypertension and diabetes [15,16]. Visceral adiposity has also been reported to be linked to increased exposure to metabolic syndrome [11,17,18], which is characterized by a prevalence of cardiovascular diseases [19,20], as well as diabetes mellitus [21] and cancer [22]. In addition, recent studies have determined that visceral adiposity is related to a higher prevalence of metabolic syndrome in studies that used bioelectrical impedance analysis [17,18,23,24].

Another difficulty associated with adult health problems is aging and the progressive decline in skeletal muscle mass, defined as sarcopenia [25]. Sarcopenia has also been linked in various studies to exposure to risks associated with metabolic syndrome [26]. These difficulties are associated, among other things, with increased insulin resistance and adherence to diseases such as diabetes mellitus and metabolic syndrome [27]. This loss of muscle mass limits the daily activities of the population, triggering other conditions associated with other morbidities such as chronic diseases [28], causing disability [29] and thus increasing medical expenses and healthcare costs [30] and even mortality [31].

Other studies have analyzed the relationship between early adult obesity (age 50) and sarcopenic obesity in older adults (age 69), demonstrating that the risk of sarcopenic obesity in older adults is preceded by obesity in early adulthood [32]. Some prospective cohort meta-analytic studies have reported an increase in mortality risk of up to 24% when sarcopenic obesity is acquired, regardless of geographical context and intervention time [33], while another meta-analysis reported that sarcopenic obesity is one of the most significant predictors of mortality in adulthood, especially if hospitalized [34].

For their part, other studies have analyzed the relationship between visceral adipose tissue mass and sarcopenic obesity in overweight and obese populations, concluding that it is necessary to look beyond BMI and consider visceral adiposity as an indicator for assessing health in adults [35]. Therefore, assessing visceral adiposity is essential to prevent metabolic risks associated with the acquisition and development of sarcopenia [36], especially because adipose tissue increases as people age [37]. It has also been reported that there is a 1.9 times greater risk of cardiovascular problems when sarcopenia is present, and when combined with visceral adiposity, the risk increases sixfold [38].

Recent studies on the use of machine learning (ML) models that analyzed visceral fat values to assess the risk of sarcopenia showed that indicators such as relative fat mass, triglyceride index, insulin resistance, and metabolic adiposity score had positive relationships with sarcopenic risk, with weight-adjusted waist index being the most significant indicator [39]. This demonstrates the importance of implementing ML to promote predictive risk models in different population groups. This is because the clinical implications of the relationships between visceral obesity and sarcopenia in young populations with NWO are not entirely clear. Thus, the objective of the present study was to examine the prevalence and metabolic profile of the NWO phenotype in a large cohort of young adults.

## 2. Materials and Methods

### 2.1. Study Design and Participants

A retrospective cross-sectional observational study was conducted using a database of 4793 body composition records obtained by bioelectrical impedance analysis (BIA) (InBody 770, InBody Co., Ltd., Seoul, Republic of Korea). Records were compiled from routine body composition assessments performed at the Nutritional Assessment and Nutritional Care Laboratory of the Tonalá University Center (University of Guadalajara, Guadalajara, Mexico) between 2018 and 2023. Participants were university students, staff and community volunteers who attended the laboratory on a self-referred or institutionally referred basis for non-clinical body composition evaluation; no advertising-based recruitment or population sampling frame was used, and the dataset therefore represents a convenience sample rather than a probability sample of the general young-adult population. No remuneration was provided.

### 2.2. Bioelectrical Impedance Analysis (BIA)

Anthropometric and body composition assessments were performed following standardized protocols to minimize measurement error. Height was determined using a portable stadiometer (SECA^®^ model 213, Hamburg, Germany) with an accuracy of 0.1 cm, with participants standing in the Frankfurt plane. For the analysis of body compartments, an eight-tactile electrode multifrequency BIA system (InBody^®^ 770, Seoul, Republic of Korea, firmware Lookin’Body) was used. This equipment uses direct segmental measurement technology, whose validity has been documented with extremely low standard estimation errors: 0.77% to 0.99% for fat percentage and 0.58 to 0.84 kg for fat-free mass (FFM) [40]. Body weight and derived parameters were automatically processed using LookinBody software (v. 120, InBody Co., Ltd., Seoul, Republic of Korea). Standardization conditions for BIA were as follows: participants were assessed in the morning after an overnight fast of at least 8–12 h; they were asked to refrain from vigorous physical exercise for at least 12 h, and from alcohol and caffeine for at least 24 h prior to the measurement; participants emptied their bladder immediately before testing; metal objects and jewellery were removed; and measurements were performed with light clothing and without shoes or socks. Participants stood on the device in the upright position with arms slightly abducted to avoid skin-to-skin contact between limbs and trunk. Because hydration status is a well-known source of variability in BIA-based estimates, participants were instructed to follow their usual fluid intake but to avoid overhydration or dehydration in the hours prior to the test; however, euhydration was not objectively confirmed through urine specific gravity or osmolality, and this is acknowledged as a methodological limitation. Visceral adiposity is reported throughout the study as VFA (in cm^2^) as estimated by the InBody algorithm, and not as “visceral fat level” (a unitless index); the two quantities are related but are not interchangeable. In women, the phase of the menstrual cycle during assessment was not recorded and could not be controlled for, which is acknowledged in the limitations.

### 2.3. Phase Angle Analysis (ϕ)

The phase angle (PhA) was used as an indicator of cell membrane integrity and tissue vitality. This parameter is derived from the relationship between resistance (*R*), which represents the opposition of body fluids to current flow, and reactance (*Xc*), which reflects the capacitance of cell membranes. Biophysically, ϕ was calculated from impedance values obtained at a frequency of 50 kHz using the following formula:

(1) $$\varphi =\arctan\left(\frac{X_{c}}{R}\right)\times \left(\frac{180}{\pi }\right)$$

This marker allows cell quality to be assessed independently of fat mass prediction equations [41].

### 2.4. Inclusion and Exclusion Criteria

Only young adults aged 18 to 35 were included (*n* = 4793), comprising men (*n* = 1625) and women (*n* = 3168). Records with incomplete data on key variables (weight, height, PBF, BMI) or biologically implausible values were excluded. In addition, records were excluded whenever the evaluation form indicated any of the following conditions known to either contraindicate BIA or to substantially bias its estimates: current or self-reported pregnancy and lactation; the presence of an implanted cardiac pacemaker, defibrillator or any other active electronic implant; ongoing use of diuretic therapy at the time of the assessment; clinically evident peripheral or generalized edema; a known diagnosis of advanced chronic kidney disease (stage ≥ 3) or chronic liver disease with ascites. Records in which any of these conditions could not be ruled out were also discarded.

### 2.5. Group Classification

Participants were stratified into four phenotypes according to the WHO criteria for BMI and clinical adiposity cut-off points for BFP [42]:

1. Underweight: BMI < 18.5 kg/m^2^
2. Healthy weight (HW): BMI 18.5–24.9 kg/m^2^ with normal BFP (≤30% in women, ≤20% in men).
3. NWO: BMI 18.5–24.9 kg/m^2^ with high BFP (≥30% in women, ≥20% in men).
4. Obesity: BMI > 25.0 kg/m^2^ with high BFP (≥ 30% in women, ≥ 20% in men).

The BFP cut-offs adopted to define NWO (≥30% in women and ≥20% in men within the normal-BMI range) are the most widely used thresholds in the NWO literature and correspond to the sex-specific upper reference limits reported by Gallagher et al. [42]. These cut-offs have been applied in prior NWO prevalence studies, allowing direct comparability of our estimates with previous reports. It is acknowledged, however, that no single BFP threshold is universally accepted, and that alternative definitions (e.g., BFP > 23.1% in men > 33.3% in women, or sex- and age-specific percentiles) have been proposed. To examine the robustness of our classification, a sensitivity analysis was performed in which NWO prevalence and between-group comparisons were re-estimated using a ±2% absolute shift in the BFP thresholds; the qualitative pattern of findings (direction and clinical magnitude of differences in VFA, free-fat mass index (FFMI), appendicular skeletal muscle mass index (ASMI) and PhA between phenotypes) was preserved, and the results of this sensitivity analysis are reported in Table A1.

### 2.6. Variables Analyzed

Anthropometric: Weight, height, BMI.

Body composition: Body fat mass (BFM) in % and kg, skeletal muscle mass in % (SMM%) and kg (SMM).

### 2.7. Derived Indices

ASMI: appendicular skeletal muscle mass (kg), i.e., the sum of the lean soft-tissue masses of the four limbs as estimated by segmental BIA, divided by height squared (m^2^).
BMI: Weight (kg) divided by height squared (m^2^).
Fat mass index (FMI): BFM (kg) divided by height squared (m^2^).
FFMI: FFM adjusted for height.
Muscle mass index (MMI): Total muscle mass (kg) divided by height squared (m^2^).
PhA (º): Phase angle.
Visceral risk marker: VFA estimated by BIA (cm^2^).

### 2.8. Ethical Considerations

The study was conducted in accordance with the Declaration of Helsinki [43] and approved by the Biosafety, Research, and Ethics Committees of the University of Guadalajara (protocol code CEI062020-01). Prior to data collection, all participants voluntarily provided their informed consent following informational sessions in which the research team detailed the objectives, procedures, and scope of the study and answered questions from those involved. Given the retrospective nature of the database analysis, anonymity and confidentiality were ensured through a process of identity masking using unique alphanumeric codes. The integrity of the information was protected by storing it on electronic devices with restricted access via biometrics and digital keys, limiting the handling of raw data exclusively to the principal investigator. It should be noted that, due to the nature of the body composition assessment design, it was not possible to apply masking techniques for either participants or evaluators.

### 2.9. Statistical Analysis

Python (v.3.10) (‘pandas’, ‘scipy.stats’, and ‘numpy’ libraries) was used for data processing. The distribution of continuous variables was evaluated by visual inspection (histograms and quantile-quantile [Q-Q] plots) and the Shapiro–Wilk test; although the latter showed significant deviations (*p* < 0.001), the asymmetry and kurtosis values remained within acceptable ranges (<2.0), which, under the central limit theorem, allowed the use of robust parametric statistics. Given the heteroscedasticity confirmed by Levene’s test (*p* < 0.001) for critical variables such as PBF, VFA, and SMM, comparisons between the four phenotypes were performed using Welch’s *t*-test for independent samples, which is superior to the standard *t*-test and nonparametric alternatives in large unbalanced samples. The relationships between body composition and cellular quality variables were analyzed using Pearson’s correlation coefficient. Finally, to overcome the tendency of large sample sizes to produce trivial values, the clinical relevance of the differences was determined using Cohen’s effect size, considering small (0.2 to <0.5), medium (0.5 to <0.8), and large (≥0.8) effects [44]. Because multiple between-group comparisons were performed across a large panel of body composition variables, the resulting *p* values were corrected for multiple testing using the Benjamini–Hochberg false discovery rate (FDR) procedure, with q set at 0.05. 

## 3. Results

Table 1 summarizes the morphofunctional characteristics of the four phenotypes in men. Despite a body weight (70.4 vs. 67.0 kg) and BMI (23.5 vs. 22.1 kg/m^2^) similar to those of the HW group, subjects classified as having NWO showed a different body composition profile. Specifically, the NWO group presented greater adiposity, with an almost doubled FMI (5.49 vs. 3.18 kg/m^2^; d = 2.77) and a higher VFA (70.3 vs. 37.3 cm^2^; d = 2.45). In parallel, the NWO group showed lower values in muscle-related indices relative to the HW group, including FFMI (d = −0.70), ASMI (d = −0.81), and PhA (5.98° vs. 6.40°; d = −0.82), which was interpreted as compatible with a state of compositionally relative sarcopenia in the absence of functional confirmation. When comparing the NWO group with the overt-obesity group, the latter showed higher absolute adiposity but also higher ASMI (8.64 kg/m^2^) and PhA (6.50°).

**Figure 1:**
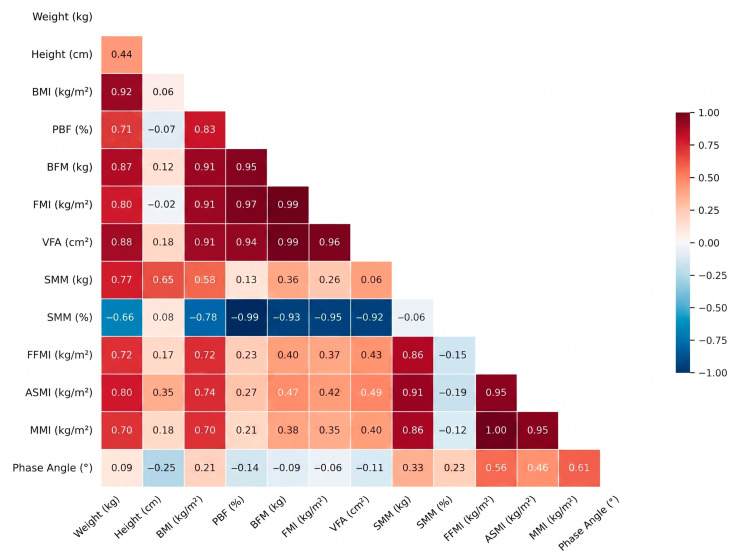
Correlation matrix (men 18–35 years). Note: ASMI: appendicular skeletal muscle mass index; BFM: body fat mass; BMI: body mass index; FFMI: fat-free mass index; FMI: fat mass index; MMI: muscle mass index; PBF: percent body fat; SMM: skeletal muscle mass; VFA: visceral fat area.

Figure 1 shows the correlation matrix for the male cohort. BFP and FMI were strongly associated with VFA (R > 0.90), and the coefficient for the BMI–VFA association (r = 0.88) was of a similar order of magnitude. A very strong negative correlation (r = −0.99) was observed between PBF and SMM%; as noted in Section 2.9, PBF and SMM% are not mathematically independent (together with the other soft-tissue compartments they sum to a fixed total), so this coefficient reflects both biological co-variation and mathematical coupling and is reported descriptively rather than as evidence of a direct tissue-level antagonism between fat and muscle. The PhA showed positive correlations with lean-mass indices (i.e., ASMI, FFMI).

Table 2 shows the women’s results. Compared to the HW group, women with NWO exhibited a substantial increase in FMI (7.87 vs. 5.38 kg/m^2^; d = 2.59) and VFA (80.2 vs. 55.9 cm^2^; d = 2.01). At the same time, a pattern compatible with a compositionally relative sarcopenia was observed in the NWO group. This phenotype presented a significantly lower SMM% (35.4% vs. 40.8%; d = −2.60) and a reduction in cell quality, evidenced by a lower PhA (5.16° vs. 5.49°; d = −0.62). When compared to the obesity group, although women with obesity had higher absolute fat levels, the NWO phenotype had a higher SMM% than women with obesity (35.4% vs. 32.9%), but lower than that of the HW and underweight groups.

**Figure 2:**
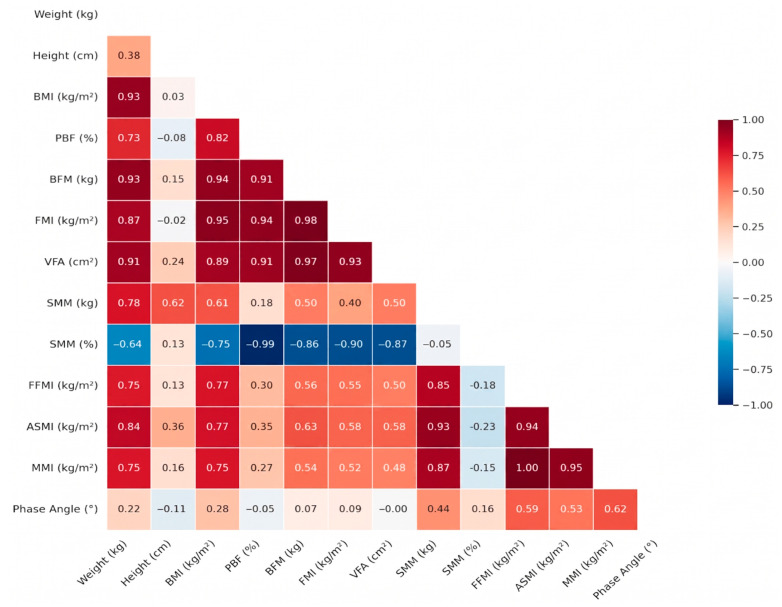
Correlation Matrix (Women 18–35 Years). Note: ASMI: appendicular skeletal muscle mass index; BFM: body fat mass; BMI: body mass index; FFMI: fat-free mass index; FMI: fat mass index; MMI: muscle mass index; PBF: percent body fat; SMM: skeletal muscle mass; VFA: visceral fat area.

In the female cohort, the correlation matrix (Figure 2) showed a pattern compatible with sex-related differences in body-composition distribution. The association between total adiposity (PBF) and VFA remained strong (r > 0.85), while the correlation between BMI and VFA was somewhat lower than in men, which is consistent with a greater relative contribution of gynoid subcutaneous fat to total body weight in women. As in the male cohort, a strong negative correlation was observed between visceral fat indices and SMM% (r = −0.87); part of this association reflects the mathematical coupling between PBF and SMM%, so the coefficient is reported descriptively and is not interpreted as evidence of a direct antagonistic effect of fat on muscle. The PhA showed positive correlations (r > 0.70) with both ASMI and FFMI.

## 4. Discussion

Our findings clearly show that relying on BMI alone fails to capture the true cardiometabolic health of young adults. In fact, it misclassified four out of ten normal-BMI participants who actually harbored excess adiposity. Despite having a normal BMI, these individuals displayed a NWO phenotype marked by a concerning clinical profile: significantly more visceral fat, less skeletal muscle mass, and a lower PhA [5,6,7].

### 4.1. From BMI-Defined Obesity to Clinical Obesity

We argue that NWO needs to be recognized as a genuine form of clinical obesity. This perspective echoes the recent Lancet Diabetes & Endocrinology Commission’s call to redefine obesity as a chronic, systemic disease where excess fat directly damages tissue and organ function [45]. Rather than being solely a preclinical warning sign, the severe visceral fat buildup, muscle depletion, and diminished PhA observed in the NWO group highlight an already established state of tissue dysfunction [13,35].

### 4.2. Visceral Adiposity, Chronic Inflammation and Early Cardiometabolic Risk

It is well established that how fat is distributed, especially when it accumulates viscerally, drives metabolic and cardiovascular issues to a greater extent than overall BMI does [11,12,37]. Even though their BMIs were nearly identical to HW peers, the NWO participants in our cohort carried substantially more visceral fat, yielding remarkably large effect sizes. This specific type of adiposity is a known driver for a host of severe conditions, from metabolic syndrome and type 2 diabetes, to coronary heart disease, hypertension, and even certain cancers [15,16,17,18,21,22].

Because NWO is frequently associated with insulin resistance, oxidative stress, and chronic low-grade inflammation, affected individuals face an elevated cardiovascular risk that completely bypasses standard BMI screening [6,7,19,20]. Consequently, these young adults are essentially living with a hidden metabolic disease, carrying a heavy risk burden that routine clinic measurements simply miss.

### 4.3. Interaction Between Visceral Adiposity and Sarcopenia: An Early Frailty Axis

A pattern compatible with a compositionally relative sarcopenia was also observed among young NWO adults. Compared with the HW, this phenotype showed lower appendicular (i.e., ASMI, MMI) and total FFMI indices together with reduced PhAs. It is important to emphasize that the term “sarcopenia” is used here in a strictly compositional sense: current operational definitions of sarcopenia such as those of the EWGSOP2 and AWGS consensus documents require the presence of reduced muscle strength and/or physical performance in addition to low muscle mass, and such functional testing (e.g., grip strength, gait speed) was not available in the present dataset. The inverse correlation observed between relative fat mass and relative skeletal muscle mass is, therefore, interpreted descriptively; as noted in Section 2.9, part of this association reflects the mathematical coupling of the compartments expressed as percentages of total body mass. Other cross-sectional and observational studies have reported, in line with our findings, that visceral fat accumulation is associated with sarcopenia and with the sarcopenic obesity phenotype across different populations [13,26,36,39], suggesting rather than proving a shared pathophysiological axis.

Looking at longitudinal data, carrying excess weight in early adulthood significantly raises the likelihood of developing sarcopenic obesity later on [32]. This is alarming, as sarcopenic obesity strongly predicts higher mortality rates, long term disability, and soaring healthcare costs [29,30,31,33,34]. It is already known that older patients facing both cardiovascular disease and sarcopenic obesity have worse clinical outcomes [38]. Therefore, the early physiological shifts observed in young NWO subjects likely represent the premature activation of this visceral adiposity–sarcopenia axis. Without an early intervention, these individuals could be on an accelerated path toward frailty and multimorbidity.

### 4.4. Phase Angle as a Biomarker of Cellular Integrity and Functional Reserve

Beyond just measuring fat and muscle, examining the PhA gave a clearer picture of actual tissue quality. The NWO group had notably lower PhA values. Because PhA correlated positively with both FFMI and ASMI, this decrease suggests a meaningful decline in cell membrane integrity and overall functional reserve. This is consistent with reference literature linking diminished PhA to malnutrition, frailty, and higher mortality risk in various clinical environments [25,31,41].

For individuals with NWO, harboring excess visceral fat alongside a low PhA acts as a warning for early cellular frailty, which may become established well before routine blood tests show any biochemical abnormalities. Fortunately, incorporating multifrequency BIA into regular check-ups can easily spot these qualitative deficits, and this may be easy to implement, as BIA is already a highly validated tool for accurately estimating body fat, fat-free mass, and visceral adiposity [14,18,23,24,40].

### 4.5. Sex Dimorphism in Fat Muscle Distribution

When the data was separated by sex, distinct distribution patterns emerged. For women, the link between BMI and visceral fat was not as strong as it was for men. This finding was biologically coherent, as gynoid subcutaneous fat heavily influences total weight in women [11,37]. Nevertheless, women classified as having NWO still presented sharply elevated FMI and visceral fat areas, alongside reduced muscle mass percentages and PhA. This reflects an intermediate, yet highly clinically significant, decline in overall body composition. Ultimately, these differences highlight why there is an urgent need for sex-specific clinical cutoffs for FMI, VFA, muscle mass, and PhA when screening for NWO and early sarcopenia. Future research and public health guidelines must approach these metrics through a sex-stratified lens [1,2,43].

### 4.6. Contrast Between Overt Obesity and NWO in Lean-Mass Indices

A notable observation in our data was the contrast between the overt-obesity and NWO phenotypes in terms of lean-mass indices. Participants in the overt-obesity group (high BMI and high total fat) showed higher appendicular lean-mass indices and higher PhAs than participants with NWO. One speculative interpretation of this pattern is that the greater mechanical loading associated with a higher body mass in overt obesity may contribute to the maintenance of appendicular muscle mass, whereas individuals with NWO who lack this mechanical stimulus while still displaying increased visceral adiposity might be more exposed to the anabolic resistance effects of low-grade systemic inflammation and ectopic lipid deposition described in the literature.

This interpretation is hypothesis-generating; the cross-sectional, compositional nature of our data does not allow causal inferences, and longitudinal studies including biochemical markers of inflammation (e.g., hs-CRP, IL-6) and functional assessments will be required to test it.

This ties into the obesity paradox often discussed in geriatric literature, in which carrying a bit of extra weight can sometimes improve survival rates provided muscle mass remains intact [4,9]. However, when high fat levels are paired with muscle depletion, mortality risk spikes dramatically [29,33,34]. The NWO phenotype observed in these young adults could very well be the early-life precursor to this exact high-risk physiological state.

### 4.7. Technology, Predictive Models and Metabolic Risk Management

Using multifrequency BIA proved invaluable for separating body compartments. It provided precise readings of visceral fat and PhA with an impressively low margin of error compared with dual-energy X-ray absorptiometry (DXA) scans [40]. It’s becoming increasingly clear that BIA-derived visceral fat metrics are excellent, practical screening tools for detecting metabolic syndrome and broader cardiometabolic risks [14,18,23,24].

At the same time, the horizon of predictive care is expanding. Machine learning models that blend visceral fat indices with anthropometric and metabolic data are showing immense promise in forecasting sarcopenia [39]. It has already been observed that technology can revolutionize patient outcomes in other fields. For example, automated insulin delivery systems can drastically improve glycemic control during the challenging postpartum period [46]. Bringing advanced body composition tools and predictive analytics into everyday practice could similarly transform how NWO can be prevented and managed in young adults.

### 4.8. Public Health and Clinical Implications

When considering an overall view of public health, the stakes are incredibly high. Global obesity rates are climbing alongside life expectancies [1,2,8]. If we continue relying on BMI as our primary screening method, the high prevalence of NWO among young people today will inevitably translate into a future burden of cardiometabolic disease, widespread disability, and overwhelming healthcare costs tomorrow [3,10,30].

Based on our findings, three steps must urgently be taken:

1. Incorporate routine body composition scans into standard check-ups for young adults, regardless of whether their BMI looks normal.
2. A shift from BMI-centered classifications toward a clinical obesity model is warranted, incorporating markers such as visceral adiposity, muscle mass, and PhA to better capture underlying body composition and metabolic risk [45].
3. Design early, targeted interventions that focus on both stripping away visceral fat and building up muscle, relying on evidence-based nutrition and structured aerobic and resistance training [27,28].

Addressing NWO early during early adulthood is not just a clinical recommendation; it is a vital window of opportunity. An early diagnosis can potentially halt the progression toward metabolic syndrome, diabetes, cardiovascular disease, and premature mortality.

### 4.9. Limitations and Future Directions

The present study has several limitations that should be acknowledged. First, the cross-sectional design precludes establishing causality between the variables under study. Second, the characteristics of our sample, primarily young adults from a specific geographical setting, limit the generalizability of our results to other populations, age groups, or contexts. Third, all body composition estimates, including total and visceral adiposity, skeletal muscle mass, appendicular skeletal muscle mass, and PhA, were derived from multifrequency BIA using the InBody 770 device. While BIA is a well-validated and highly reproducible method in young, healthy, euhydrated adults [40], and has shown good correlation with visceral fat area measured by computed tomography in previous research [47,48], it is not the gold standard for the quantification of lean mass, fat mass, or visceral adipose tissue. Reference methods such as DXA, magnetic resonance imaging (MRI), and computed tomography (CT) provide more precise direct anatomical measurements. VFA derived from BIA is known to be affected by hydration status, body position, ethnicity, and the specific prediction algorithm implemented by the device. Therefore, our estimates should be interpreted as operational body-composition indicators obtained under standardized conditions, rather than as direct anatomical measurements. Fourth, the dataset did not include biochemical markers of metabolic and inflammatory status (e.g., fasting glucose, fasting insulin, homeostatic model assessment of insulin resistance (HOMA-IR), lipid profile (total cholesterol, low-density lipoprotein cholesterol [LDL-c], high-density lipoprotein cholesterol [HDL-c], and triglycerides, high-sensitivity C-reactive protein (hs-CRP), interleukin-6 (IL-6), leptin, and adiponectin). Consequently, the present work cannot directly document the cardiometabolic or inflammatory phenotype of the NWO group. Its characterization as a “high-risk phenotype” rest on extrapolation from prior literature rather than on biochemical evidence obtained in this specific cohort. Fifth, consistent with the terminological change adopted throughout the manuscript, functional assessments of muscle performance (e.g., handgrip strength, dynamometry, chair-rise test, or gait speed) were not available. For this reason, the term sarcopenia is used here strictly in its compositional sense (low relative lean-mass indices), and the current European Working Group on Sarcopenia in Older People 2 (EWGSOP2) and Asian Working Group for Sarcopenia (AWGS) operational definitions of sarcopenia (which require reduced muscle strength and/or physical performance) cannot be formally applied to our cohort. Despite these limitations, this study highlights an opportunity to further analyze the limiting factors of BMI as a predictor of metabolic health in young people. Future longitudinal, multicenter, and experimental studies are needed to confirm whether these associations are present across different population groups, ages, and genders. Such studies should combine BIA- or DXA-based body composition with rigorous biochemical profiling and standardized functional testing to establish whether the compositional pattern described here translates into measurable metabolic and functional impairment.

## 5. Conclusions

The present study shows that, in this cohort of young adults, BMI alone failed to identify excess adiposity in approximately 4 out of 10 normal-BMI participants. The NWO phenotype, as operationalized here, was characterized by higher visceral fat area, lower relative skeletal muscle mass indices and lower PhA compared with the HW group, a pattern compatible with compositionally relative sarcopenia in the absence of functional confirmation. Interestingly, within our sample, participants with NWO also showed lower PhA and lower appendicular lean-mass indices than participants classified as overtly obese; this observation is hypothesis-generating and, given the cross-sectional and BIA-based nature of our data, should not be interpreted as evidence that “hidden obesity” is biologically more aggressive to muscle tissue than overt obesity. From a public health perspective, these findings support the view that body composition assessment using BIA or equivalent techniques may provide relevant complementary information to BMI in routine evaluations of young adults, particularly with respect to visceral adiposity and lean-mass indices. Longitudinal studies that incorporate biochemical markers of metabolic and inflammatory status and standardized functional testing are needed to establish the clinical significance of the NWO phenotype and its relevance for primary prevention of cardiometabolic disease.

## Figures and Tables

**Table 1:** Comparison of anthropometric profile, body composition indices, and markers of cellular quality in young men stratified by BMI and adiposity phenotypes.

Variable	Underweight (*n* = 31)	HW (*n* = 516)	NWO (*n* = 159)	Obesity (*n* = 919)	HW vs. NWO *p* Value	Cohen’s d	O vs. NWO *p* Value	Cohen’s d	U vs. NWO *p* Value	Cohen’s d
Weight (kg)	53.4 ± 5.1	67.0 ± 7.0	70.4 ± 6.2	88.8 ± 13.0	<0.001	0.50	<0.001	1.51	<0.001	2.81
Height (cm)	177.6 ± 5.6	174.2 ± 6.1	173.1 ± 5.7	175.0 ± 6.6	0.031	−0.19	<0.001	0.29	<0.001	−0.80
BMI (kg/m^2^)	16.9 ± 1.3	22.1 ± 1.8	23.5 ± 1.2	29.0 ± 3.4	<0.001	0.85	<0.001	1.71	<0.001	5.42
PBF (%)	9.9 ± 1.9	14.3 ± 3.3	23.4 ± 2.5	27.3 ± 7.4	<0.001	2.87	<0.001	0.56	<0.001	5.54
BFM (kg)	5.3 ± 1.0	9.6 ± 2.7	16.5 ± 2.2	24.7 ± 9.5	<0.001	2.64	<0.001	0.93	<0.001	5.41
FMI (kg/m^2^)	1.67 ± 0.31	3.18 ± 0.87	5.49 ± 0.70	8.07 ± 3.10	<0.001	2.77	<0.001	0.90	<0.001	5.84
VFA (cm^2^)	17.9 ± 6.6	37.3 ± 14.2	70.3 ± 10.8	102.8 ± 36.4	<0.001	2.45	<0.001	0.96	<0.001	5.13
SMM (kg)	26.7 ± 3.1	32.5 ± 3.5	30.3 ± 3.1	36.5 ± 4.8	<0.001	−0.66	<0.001	1.37	<0.001	1.15
SMM (%)	50.0 ± 1.6	48.5 ± 2.0	42.9 ± 1.6	41.4 ± 4.4	<0.001	−2.87	<0.001	−0.37	<0.001	−4.35
FFMI (kg/m^2^)	15.2 ± 1.3	18.9 ± 1.4	18.0 ± 1.0	20.9 ± 1.7	<0.001	−0.70	<0.001	1.82	<0.001	2.59
ASMI (kg/m^2^)	6.77 ± 0.53	7.86 ± 0.53	7.44 ± 0.45	8.64 ± 0.68	<0.001	−0.81	<0.001	1.84	<0.001	1.44
MMI (kg/m^2^)	8.45 ± 0.80	10.69 ± 0.85	10.07 ± 0.64	11.89 ± 1.05	<0.001	−0.77	<0.001	1.82	<0.001	2.42
PhA (°)	5.49 ± 0.63	6.40 ± 0.53	5.98 ± 0.48	6.50 ± 0.54	<0.001	−0.82	<0.001	0.99	<0.001	0.97

Note: Data are presented as mean ± standard deviation. Abbreviations: ASMI: appendicular skeletal muscle mass index; BFM: body fat mass; BMI: body mass index; FFMI: fat-free mass index; FMI: fat mass index; HW: healthy weight; MMI: muscle mass index; NWO: normal weight obesity; O: obesity; PBF: percentage of body fat; PhA: phase angle; SMM: skeletal muscle mass; U: underweight; VFA: visceral fat area.

**Table 2:** Comparison of anthropometric profile, body composition indices, and markers of cellular quality in young women stratified by BMI and adiposity phenotypes.

Variable	Underweight (*n* = 218)	HW (*n* = 963)	NWO (*n* = 853)	Obesity (*n* = 1134)	HW vs. NWO *p* Value	Cohen’s d	O vs. NWO *p* Value	Cohen’s *d*	U vs. NWO *p* Value	Cohen’s *d*
Weight (kg)	44.7 ± 4.4	55.2 ± 5.9	58.5 ± 5.9	75.5 ± 12.8	<0.001	0.56	<0.001	1.63	<0.001	2.46
Height (cm)	160.2 ± 6.9	161.2 ± 5.9	159.9 ± 5.9	160.7 ± 6.0	<0.001	−0.21	0.003	0.13	0.557	−0.05
BMI (kg/m^2^)	17.4 ± 1.0	21.2 ± 1.7	22.8 ± 1.5	29.2 ± 4.2	<0.001	1.04	<0.001	1.90	<0.001	3.89
PBF (%)	22.8 ± 4.6	25.3 ± 3.6	34.4 ± 2.8	40.1 ± 6.1	<0.001	2.76	<0.001	1.16	<0.001	3.53
BFM (kg)	10.2 ± 2.2	14.0 ± 2.6	20.1 ± 2.8	30.8 ± 9.4	<0.001	2.29	<0.001	1.46	<0.001	3.72
FMI (kg/m^2^)	3.99 ± 0.90	5.38 ± 0.97	7.87 ± 0.95	11.88 ± 3.44	<0.001	2.59	<0.001	1.50	<0.001	4.13
VFA (cm^2^)	43.1 ± 10.0	55.9 ± 11.8	80.2 ± 12.4	112.9 ± 27.2	<0.001	2.01	<0.001	1.48	<0.001	3.11
SMM (kg)	18.4 ± 2.4	22.5 ± 2.9	20.7 ± 2.4	24.6 ± 3.3	<0.001	−0.67	<0.001	1.31	<0.001	0.97
SMM (%)	41.1 ± 2.9	40.8 ± 2.3	35.4 ± 1.8	32.9 ± 3.5	<0.001	−2.60	<0.001	−0.87	<0.001	−2.81
FFMI (kg/m^2^)	13.4 ± 0.8	15.8 ± 1.3	15.0 ± 1.0	17.3 ± 1.5	<0.001	−0.75	<0.001	1.79	<0.001	1.67
ASMI (kg/m^2^)	5.21 ± 0.48	6.26 ± 0.58	5.93 ± 0.48	7.00 ± 0.69	<0.001	−0.61	<0.001	1.74	<0.001	1.50
MMI (kg/m^2^)	7.14 ± 0.52	8.64 ± 0.80	8.08 ± 0.62	9.49 ± 0.92	<0.001	−0.78	<0.001	1.75	<0.001	1.56
PhA (°)	4.82 ± 0.47	5.49 ± 0.56	5.16 ± 0.47	5.55 ± 0.60	<0.001	−0.62	<0.001	0.71	<0.001	0.72

Note: Data are presented as mean ± standard deviation. Abbreviations: ASMI: appendicular skeletal muscle mass index; BFM: body fat mass; BMI: body mass index; FFMI: fat-free mass index; FMI: fat mass index; HW: healthy weight; MMI: muscle mass index; NWO: normal weight obesity; O: obesity; PBF: percentage of body fat; PhA: phase angle; SMM: skeletal muscle mass; U: underweight; VFA: visceral fat area.

## Data Availability

The raw data supporting the conclusions of this article will be made available by the authors upon request.

## Appendix A

**Table A1:** Sensitivity analysis of differences in body composition between phenotypes using alternative adiposity thresholds.

Variable	Scenario (Shift)	HW (Mean)	NWO (Mean)	*p* Value	Cohen’s *d*
**Men (*n* = 1625)**					
VFA (cm^2^)	Base (20%)	37.0	70.0	<0.001	−2.48
	+2% Shift (22%)	39.6	74.3	<0.001	−2.36
	−2% Shift (18%)	33.9	65.3	<0.001	−2.56
ASMI (kg/m^2^)	Base (20%)	7.9	7.5	<0.001	0.78
	+2% Shift (22%)	7.8	7.4	<0.001	0.92
	−2% Shift (18%)	7.9	7.6	<0.001	0.62
FFMI (kg/m^2^)	Base (20%)	18.9	18.0	<0.001	0.66
	+2% Shift (22%)	18.8	17.7	<0.001	0.86
	−2% Shift (18%)	18.9	18.2	<0.001	0.50
**Women (*n* = 3168)**					
VFA (cm^2^)	Base (30%)	55.8	80.1	<0.001	−2.01
	+2% Shift (32%)	58.2	83.0	<0.001	−2.02
	−2% Shift (28%)	52.3	76.6	<0.001	−1.98
ASMI (kg/m^2^)	Base (30%)	6.3	5.9	<0.001	0.61
	+2% Shift (32%)	6.2	5.9	<0.001	0.60
	−2% Shift (28%)	6.3	6.0	<0.001	0.61
FFMI (kg/m^2^)	Base (30%)	15.8	15.0	<0.001	0.75
	+2% Shift (32%)	15.7	14.9	<0.001	0.73
	−2% Shift (28%)	15.9	15.1	<0.001	0.72

Note: NWO: normal weight obesity; HW: healthy weight; VFA: visceral fat area; ASMI: appendicular skeletal muscle mass index; FFMI: fat-free mass index. Base scenarios for percent body fat (PBF) thresholds were ≥20% for men and ≥30% for women. Sensitivity scenarios applied a ±2% absolute shift to these thresholds. *p* values calculated via Welch’s *t*-test.
